# An *in vitro* assay of collagen fiber alignment by acupuncture needle rotation

**DOI:** 10.1186/1475-925X-7-19

**Published:** 2008-07-07

**Authors:** Margaret Julias, Lowell T Edgar, Helen M Buettner, David I Shreiber

**Affiliations:** 1 Department of Chemical and Biochemical Engineering, Rutgers, The State University of New Jersey, Piscataway, NJ, USA; 2 Department of Biomedical Engineering, Rutgers, The State University of New Jersey, Piscataway, NJ, USA

## Abstract

**Background:**

During traditional acupuncture therapy, soft tissues attach to and wind around the acupuncture needle. To study this phenomenon in a controlled and quantitative setting, we performed acupuncture needling in vitro.

**Methods:**

Acupuncture was simulated in vitro in three-dimensional, type I collagen gels prepared at 1.5 mg/ml, 2.0 mg/ml, and 2.5 mg/ml collagen, and either crosslinked with formalin or left untreated. Acupuncture needles were inserted into the gels and rotated via a computer-controlled motor at 0.3 rev/sec for up to 10 revolutions while capturing the evolution of birefringence under cross-polarization.

**Results:**

Simulated acupuncture produced circumferential alignment of collagen fibers close to the needle that evolved into radial alignment as the distance from the needle increased, which generally matched observations from published tissue explant studies. All gels failed prior to 10 revolutions, and the location of failure was near the transition between circumferential and radial alignment. Crosslinked collagen failed at a significantly lower number of revolutions than untreated collagen, whereas collagen concentration had no effect on gel failure. The strength of the alignment field increased with increasing collagen concentration and decreased with crosslinking. Separate studies were performed in which the gel thickness and depth of needle insertion were varied. As gel thickness increased, gels failed at fewer needle revolutions. For the same depth of insertion, alignment was greater in thinner gels. Alignment increased as the depth of insertion increased.

**Conclusion:**

These results indicate that the mechanostructural properties of soft connective tissues may affect their response to acupuncture therapy. The in vitro model provides a platform to study mechanotransduction during acupuncture in a highly controlled and quantitative setting.

## Background

In traditional acupuncture therapy, fine needles are inserted into the skin at specific points on the body and manipulated manually, typically by needle rotation. During this process, it is important to achieve the characteristic of "de qi", a physical sensation experienced by the patient, and a biomechanical phenomenon experienced by the acupuncture therapist that is also known as needle grasp. In needle grasp, the therapist feels a resistance to further needle manipulation, which has been described as a fish biting on a line (see [1]). Recent studies by Langevin et al suggest that needle grasp results when collagen fibers of the loose, subcutaneous connective tissue couple to and wind around the rotating needle [2-4]. The connective tissue experiences significant deformation during this process. In addition, acupuncture needle manipulation in connective tissue explants induces cytoskeletal remodeling by fibroblasts [5,6], the predominant cell type in loose connective tissue, supporting the hypothesis that tissue deformation due to needle manipulation mechanically stimulates fibroblasts, resulting in mechanotransduction effects that may contribute to therapeutic benefits [2,3].

These in vivo and ex vivo findings have pioneered an exciting focus on connective tissue involvement in acupuncture, but also raise interesting questions about acupuncture needling and collagen fiber winding that motivate the need for in vitro tools. From a therapeutic perspective, if fiber winding does play an important role then parameters that affect winding would be expected to alter the therapeutic response. Candidate parameters include ones that would affect tissue mechanics, such as matrix composition, density, and stiffness, as well as the thickness of the tissue layer(s). Identifying relationships among tissue properties, winding, and therapeutic effects through in vivo experiments alone, however, is complicated by normal in vivo variations in tissue properties. For example, the collagen content in skin is known to differ with location, tissue type, and skin layer [7-9]. Additionally, collagen content decreases with aging [10-12], potentially due to an increase in collagen crosslinking [13], and the thickness of the subcutaneous connective tissue is variable in humans, which may also affect the response to needling [14]. Accounting for these variations significantly increases the sample number required to obtain statistically meaningful in vivo data.

With this in mind, we have developed an in vitro approach to examine the first part of the proposed therapeutic mechanism, i.e., the link between tissue properties and collagen fiber winding, which can be used to guide further in vivo investigation. Specifically, we used type I collagen gels to study the effects of matrix properties on collagen fiber response to acupuncture needle rotation. We subjected gels with different collagen concentrations and degrees of crosslinking to computer-controlled needle rotation and used polarized light imaging to monitor the change in collagen fiber alignment during needle rotation. In separate gels, we varied the thickness and depth of needle insertion. Our results demonstrate that the winding response of fibrillar collagen to needle rotation resembles that of loose connective tissue and varies with network density and stiffness and the depth of needle insertion. The quantitative approach developed in this work provides a useful new tool to aid in elucidating tissue-level mechanisms of acupuncture.

## Methods

### Collagen gel preparation

Acellular collagen gels were prepared from lyophilized collagen (Elastin Products, Owensville, MO) as previously described [15]. A stock solution was prepared by dissolving 3.75 mg/ml collagen in 0.02 N acetic acid. The stock solution was diluted with 0.02 N acetic acid, neutralized with 0.1 N NaOH, and further diluted with M199 and 10XMEM media (Sigma Aldrich, St Louis, MO) to achieve the desired final collagen concentration of 1.5, 2.0, or 2.5 mg/ml (see below). A 3-ml gel solution was poured into a 35-mm glass bottom MatTek dish with a 20-mm glass microwell (MatTek Corporation) and incubated at 37°C for 2 hr to ensure complete fibril formation. After self assembly, 1 ml of fresh phosphate buffered saline (PBS) was added on top of the gel. In some samples, the collagen solution was spiked with FITC-labeled collagen (Elastin Products, Owensville, MO) in a 1:9 ratio of fluorescent collagen:collagen to permit visualization of collagen fibers using confocal microscopy. For crosslinking studies, gels were incubated in 1 ml of 10% neutral buffered formalin solution for 15 min on a rocker plate. The formalin solution was then replaced with PBS. The final height of gel was 4 mm and did not vary significantly with crosslinking.

### In vitro acupuncture

A computer-controlled motor (MicroMo Electronics, Inc.) was used to needle the collagen gels. A 250-μm stainless steel acupuncture needle (Seirin, Tokyo, Japan) was attached to the motor and inserted perpendicular to the surface of the gel to a depth of 3 mm using a calibrated micromanipulator. The needle was rotated at 0.3 rev/s for either 2 or 4 revolutions in samples used for confocal imaging, or for 10 revolutions in samples used for continuous recording of the evolution of fiber alignment with polarized light microscopy.

A separate series of experiments was performed to investigate the influence of the depth of needle insertion on the response of the gel. Collagen solution was prepared at 2.0 mg/ml as described above, and gels were prepared such that the final gel height at the middle of the gel was 2 mm, 4 mm, or 6 mm. These gels were subjected to in vitro acupuncture as described above, with the depth of needle insertion varying from 1.5 mm to 4.5 mm and 25%–75% of the thickness of the gel (Table 1).

**Table 1:** Conditions for investigating effects of needle insertion depth

Gel Height (mm)	Insertion Depth (mm)	Insertion Depth/Gel Height (%)
2	1.50	75.0
4	1.50	37.5
4	2.25	56.3
4	3.00	75.0
6	1.50	25.0
6	2.25	37.5
6	3.00	50.0
6	4.50	75.0

### Confocal imaging

Confocal microscopy was used to examine the fibril alignment pre- and post- needle rotation. The MatTek dish was covered with a 3 mm-thick sheet of poly(dimethyl siloxane) (PDMS), through which the acupuncture needle was inserted prior to entering the gel. The needle was rotated either 2 or 4 revolutions with the motor, after which the needle was de-coupled from the motor. Insertion through the PDMS prevented the needle from recoiling when detached from the motor and allowed the transfer of the dish to the confocal microscope. The gel was imaged with a 63× objective using a laser wavelength of 488 nm to visualize the fluorescent collagen fibers (excitation 497 nm, emission 520 nm). The confocal images were compared to bright field and polarized light images taken during the needling procedure.

### Polarized light imaging

**Figure 1:**
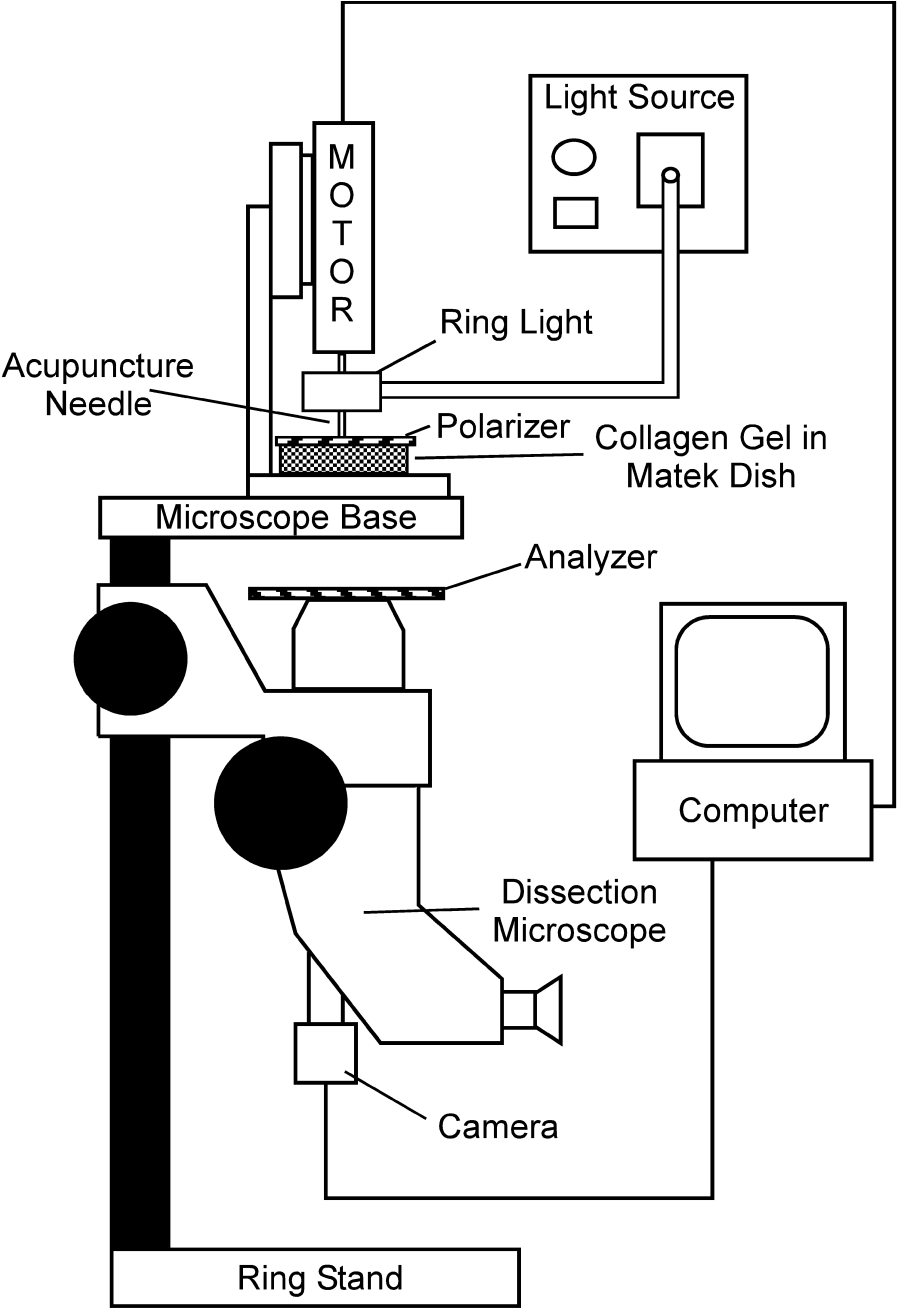
Schematic of polarized light microscopy system. A dissection stereomicroscope with a USB camera was mounted upside-down to a bench top. A fiber-optic ring light was attached to the motor housing providing a light source to the sample without hindrance from the motor. The polarizer was placed on top of the sample dish, and the analyzer was placed on the microscope as shown with the axis of polarization orthogonal to the axis for the polarizer. A small hole in the polarizer allowed free insertion and rotation of the acupuncture needle in the sample.

Polarization light microscopy (PLM) was used to observe and image the evolution of fiber alignment continuously in real time. A dissection stereomicroscope (Carl Zeiss Microimaging, Thornwood, NY) with a USB camera (Matrix Vision, GmbH, Oppenweiler, Germany) was physically inverted and clamped to a benchtop, such that the base of the microscope provided a platform to hold the motor stand and MatTek dish (Figure 1). A fiber-optic ring light (Edmund Optics, Barrington, NJ) was attached to the motor housing. The gel was placed between two polarizers, which were positioned as 'cross-polars' with their respective angles of polarization 90° apart. In this arrangement, as the light passes through the filter-sample-filter optic train, the darkest area of the resulting image occurs where collagen fibers are oriented parallel or perpendicular to the optical axis of either polarizing element; the brightest area occurs where collagen fibers are oriented 45° to the filter optical axis. The collagen gel was needled for 10 revolutions. Images were captured at 6 frames per second during needle rotation and were analyzed with MATLAB (The Mathworks, Inc, Natick, MA), as described below. Six experiments, each comprising three replicates, were performed for untreated collagen gels, and five experiments were performed for crosslinked gels, also in triplicate.

### Image analysis

**Figure 2:**
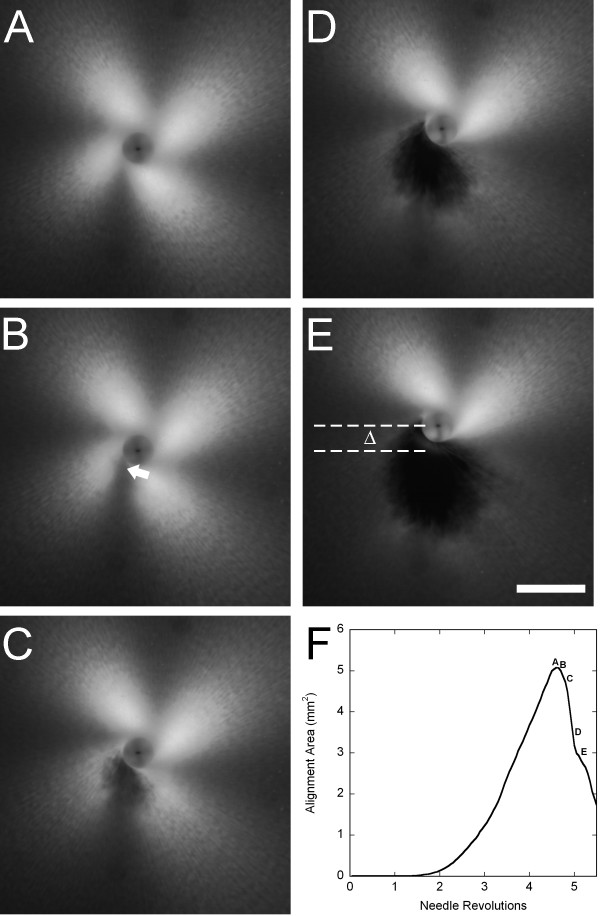
Winding and failure of collagen gels during in vitro acupuncture. (A) PLM image of the gel immediately before the onset of tearing. The characteristic '4-leaf clover' pattern of birefringence increases in size up to the point of failure as the gel becomes increasingly aligned due to winding around the needle. (B-E) Development of gel failure at 0.5 sec (0.15 rev) intervals. At the onset of tearing (B), a weakening of the birefringence can be observed near the needle where the dense, circumferentially wound center transitions to radially aligned fibers (arrow). As failure ensues, a hole is observed in the gel (C-E), and the residual stress in the remainder of the gel is enough to bend the needle, as indicated by the shift in needle position, Δ, directed away from the tear. The increasing size of the tear results in a decreasing area of birefringence. (F) Images A-E marked on a plot of the area above a threshold intensity vs. needle revolutions. The peak represents the image taken at maximum alignment immediately prior to the onset of failure. Bar: 1 mm.

In all cases, PLM generated images with a '4-leaf clover' morphology of birefringence that emerged and increased in area with increasing needle rotation, extending beyond the field of the captured image, until gel failure, at which point the intensity decreased (Figure 2). The PLM images were imported into MATLAB to quantify the birefringence. First, the failure point for each individual experiment was identified by plotting the number of pixels with intensity greater than a given threshold value, determined as described below, against the number of needle revolutions and identifying the global maximum of the curve (Figure 2F). This point was confirmed by visual inspection of the image sequence. Failure points were compared statistically using a two-way ANOVA, with collagen concentration and crosslinking as fixed effects (SPSS 12.0, Chicago, IL). Significance levels were set at *P *< 0.05. The earliest failure point (rounded down to the nearest integer) among all experiments was 4 revolutions for untreated samples and 2 revolutions for crosslinked samples.

The evolution of birefringence with needle rotation, reflecting the increase in fiber alignment, was quantified by determining a continuous index of the area of alignment. To apply a consistent criterion for comparing alignment across different conditions, the image sequence for each experiment was first normalized by subtracting the mode intensity value of the first image, which was taken prior to needle rotation and represented the background intensity level, from all remaining images in the sequence. This removed background differences among different collagen concentrations and smoothed out minor day-to-day fluctuations.

**Figure 3:**
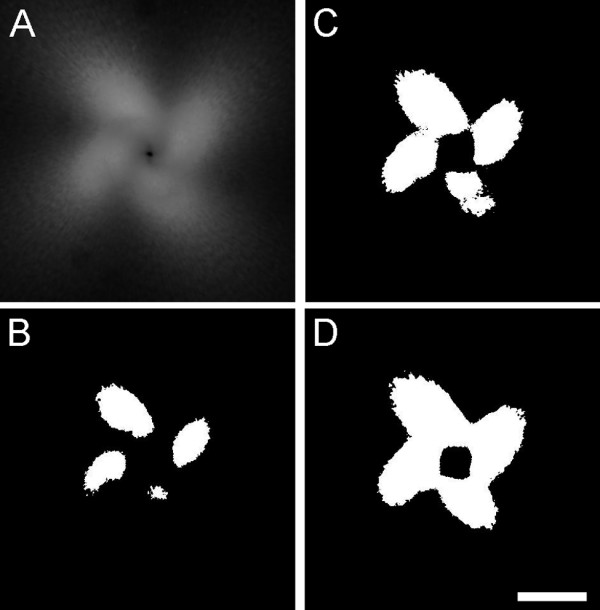
Methodology for identifying threshold criteria. From each image set, the image at 2 or 4 revolutions was extracted (A) and sequentially binarized with a decreasing grayscale value, beginning with the maximum intensity present in the image. As the threshold value decreased, the binarized image began to resemble a 4-leaf clover (B, C). The threshold value for the gel was set as the maximum grayscale value that produced a complete clover structure with no interruptions in the 4 leaves (D). Bar: 1 mm.

To determine the operating threshold intensity for each experimental set of images, the image at 2 (crosslinked samples) or 4 (untreated samples) revolutions was binarized at decreasing threshold intensity levels, beginning with the maximum intensity present in the image. As the threshold value decreased, the binarized image began to resemble a 4-leaf clover. The operating threshold value was identified as the maximum value for which a complete clover structure was observed (Figure 3). Using this threshold value, each frame in the image set was converted to a binary image, and the area of pixels greater than or equal to that intensity was calculated. Comparisons among untreated collagen gels were made up to 4 revolutions, and comparisons among crosslinked collagen gels, or between crosslinked and untreated gels, were made up to 2 revolutions, the earliest failure points observed among all experiments within these respective conditions.

### Collagen gel rheology

Parallel plate rheometry was used to assess the mechanical properties of the collagen gels. Mechanical testing was done using a Rheometrics SR-2000 parallel plate rheometer with a temperature-controlled incubation chamber maintained at 37°C (TA Instruments, New Castle, DE). A sample well was formed by punching a 25 mm diameter hole in a 4 mm thick layer of PDMS. Type I collagen solutions were prepared at concentrations of 1.5, 2.0, and 2.5 mg/ml, as described above, and 2 ml were pipetted into the well, which was then transferred to a 37°C incubator. After self assembly, the gels were carefully removed with a spatula and transferred to the bottom plate of the rheometer. The top plate was lowered to a height of 2.0 mm. The dynamic storage and loss moduli of the gels were evaluated at 1% shear strain amplitude at frequencies ranging from 0.1 – 10 Hz. Five samples were tested for each of the 6 conditions. The moduli were analyzed statistically with a two-way ANOVA. Significance levels were set at *P *< 0.05.

## Results

### General observations

**Figure 4:**
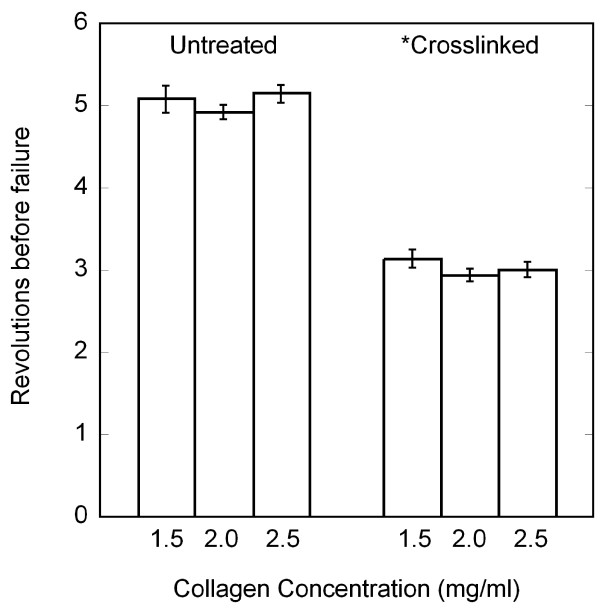
Revolutions to failure (average +/- standard error) during in vitro acupuncture. The number of revolutions before gel tearing was identified from alignment area curves and verified visually from the image sets. Crosslinking the collagen significantly decreased the ability of the collagen gels to withstand needle rotation without tearing (*, 2-way ANOVA, *P *< 0.001), whereas changing the collagen concentration had no effect (*P *= 0.274)

During continuous needle rotation, all gels exhibited tearing prior to 10 revolutions. Whereas collagen concentration did not affect the failure of the gels (*P *= 0.274), crosslinked gels failed at a significantly lower number of revolutions than untreated gels (*P *< 0.001) (two-way ANOVA) (Figure 4). Therefore, to compare different conditions, quantitative analyses were performed up to a standardized number of revolutions that represented the lowest integer number before failure among all samples.

### Collagen imaging

**Figure 5:**
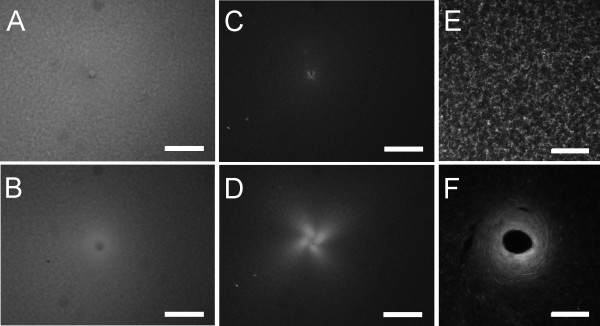
Images of 2 mg/ml fluorescently spiked collagen gels before needling and after 2 revolutions. (A) Brightfield imaging of the gel with needle inserted before needling shows a small hole in an otherwise uniform field. (B) After needle rotation, the collagen fiber density appears to change around the needle. (C) Under cross-polars, the needle hole is again evident in an otherwise random field before needle rotation. (D) After rotation, the significant bright regions indicating collagen fiber alignment 45° off-axis. (E) Using confocal microscopy, the random orientation of collagen fibers is apparent. (F) After needling, the fiber density increases around the needle. Fibers near the needle are aligned circumferentially and transition to radial alignment away from the needle. Bars: A-D, 1 mm; E-F, 50 μm.

Gel morphology before and after 2 revolutions is shown in brightfield (Figure 5A &5B), PLM (Figure 5C &5D), and confocal (Figure 5E &5F) images. Collagen fiber winding was not obvious in bright field images, but could be inferred from PLM images (see below), and was directly observed in confocal images. Prior to needle rotation, collagen fibers were randomly oriented. After needle rotation, circumferential alignment was observed close to the needle, which evolved into radial alignment as the distance from the needle increased (Figure 5F). The effect was similar but more pronounced after 4 revolutions (data not shown).

### Quantitative PLM

**Figure 6:**
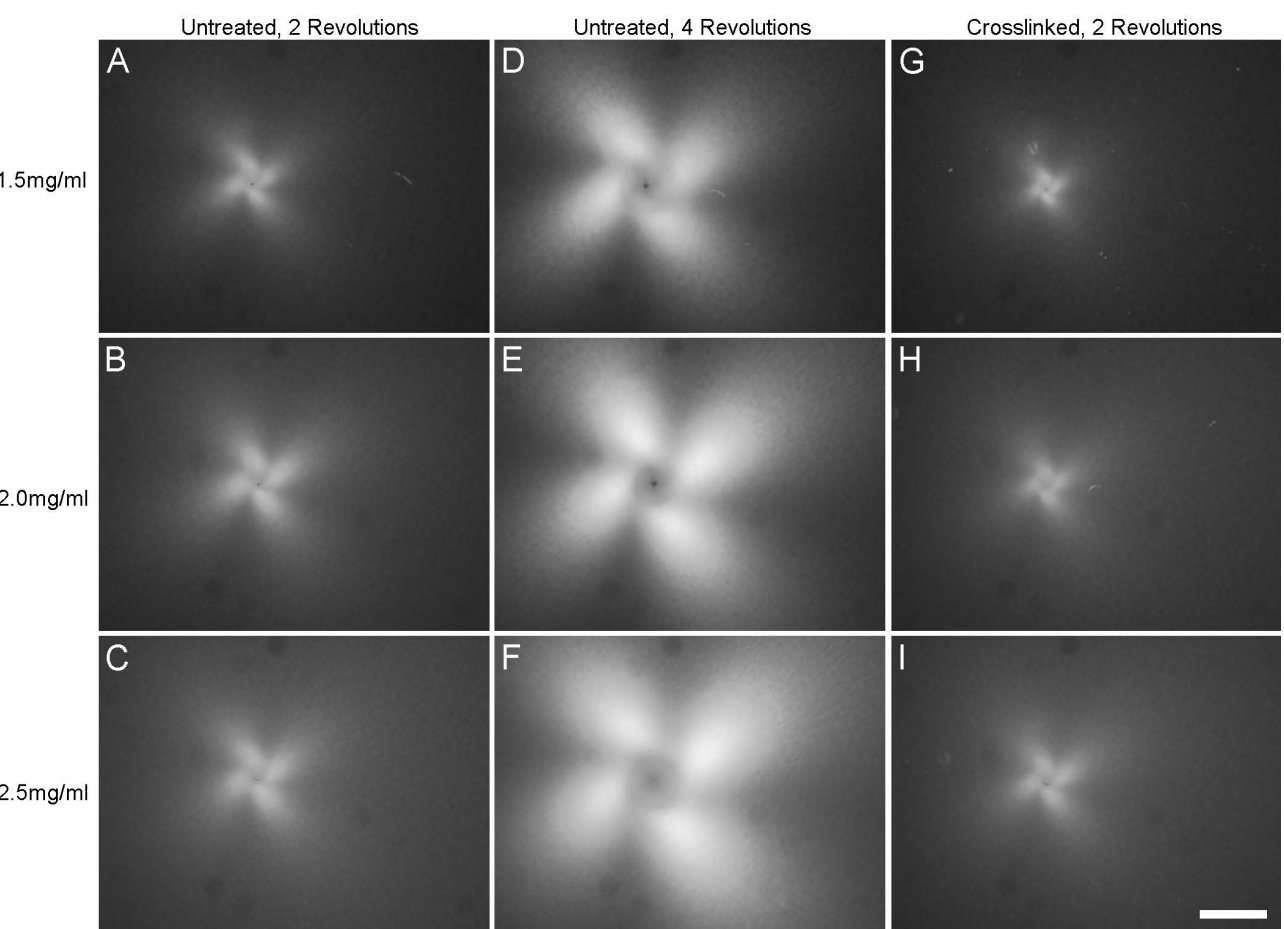
Polarized light images of collagen gel response to in vitro acupuncture after 2 needle revolutions (A-C), and 4 revolutions (D-F) in untreated collagen gels and 2 revolutions (G-I) in crosslinked gels. The birefringent area increased with increasing revolutions, and was greater in untreated collagen compared to crosslinked collagen at the same number of revolutions. This area also increased with increasing collagen concentration for each condition (A, D, G – 1.5 mg/ml; B, E, H – 2.0 mg/ml; C, F, I – 2.5 mg/ml). Bar = 1 mm.

**Figure 7:**
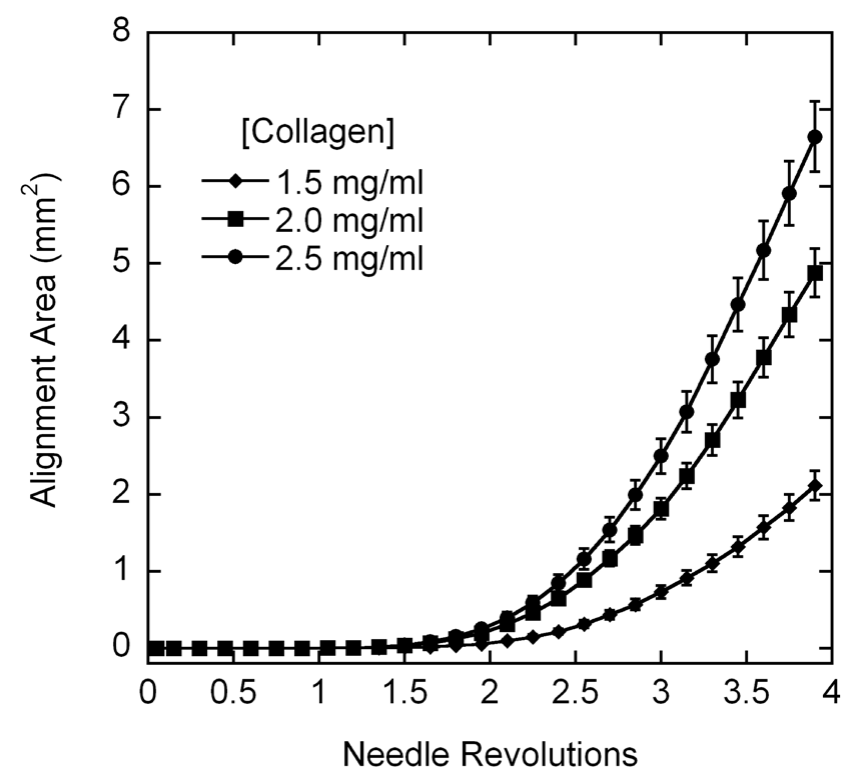
Effects of collagen concentration on area of alignment (average +/- standard error). The birefringent area of alignment was identified from image sets binarized based on the image at 4 revolutions, and plotted as a function of needle revolution. Increasing the collagen concentration increased the area.

**Figure 8:**
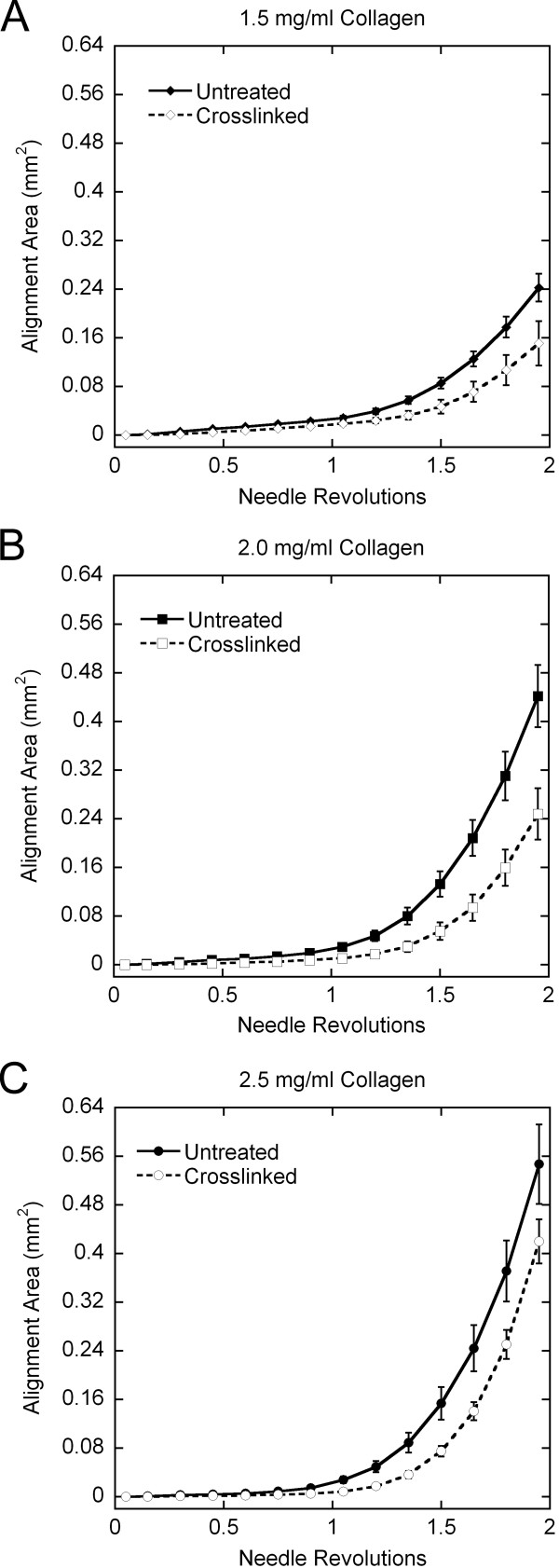
Effects of crosslinking on area of alignment (average +/- standard error). The birefringent area of alignment was identified from image sets binarized based on the image at 2 revolutions. Needle rotation in crosslinked collagen gels generated less alignment than untreated collagen gels at each collagen concentration. The area of alignment increased with increasing collagen concentration for both untreated and crosslinked collagen.

The alignment pattern observed with confocal microscopy was evident in PLM images as a clover-leaf pattern of birefringence, where fiber alignment 45° off-axis generates an intensity peak. The area of alignment at the same number of revolutions was visually greater with increasing collagen concentration and in untreated collagen gels vs. crosslinked gels (up to 2 revolutions) (Figure 6). A continuous index of the area of alignment was generated by binarizing the complete set of images in each run using a threshold intensity as described above. For untreated collagen gels, the average area of alignment increased more rapidly and to a higher final value with increasing concentration (Figure 7). Crosslinked samples aligned more gradually than untreated gels (Figure 8), and also demonstrated the trend of increased area of alignment with increasing collagen concentration.

### Depth of needle insertion

**Figure 9:**
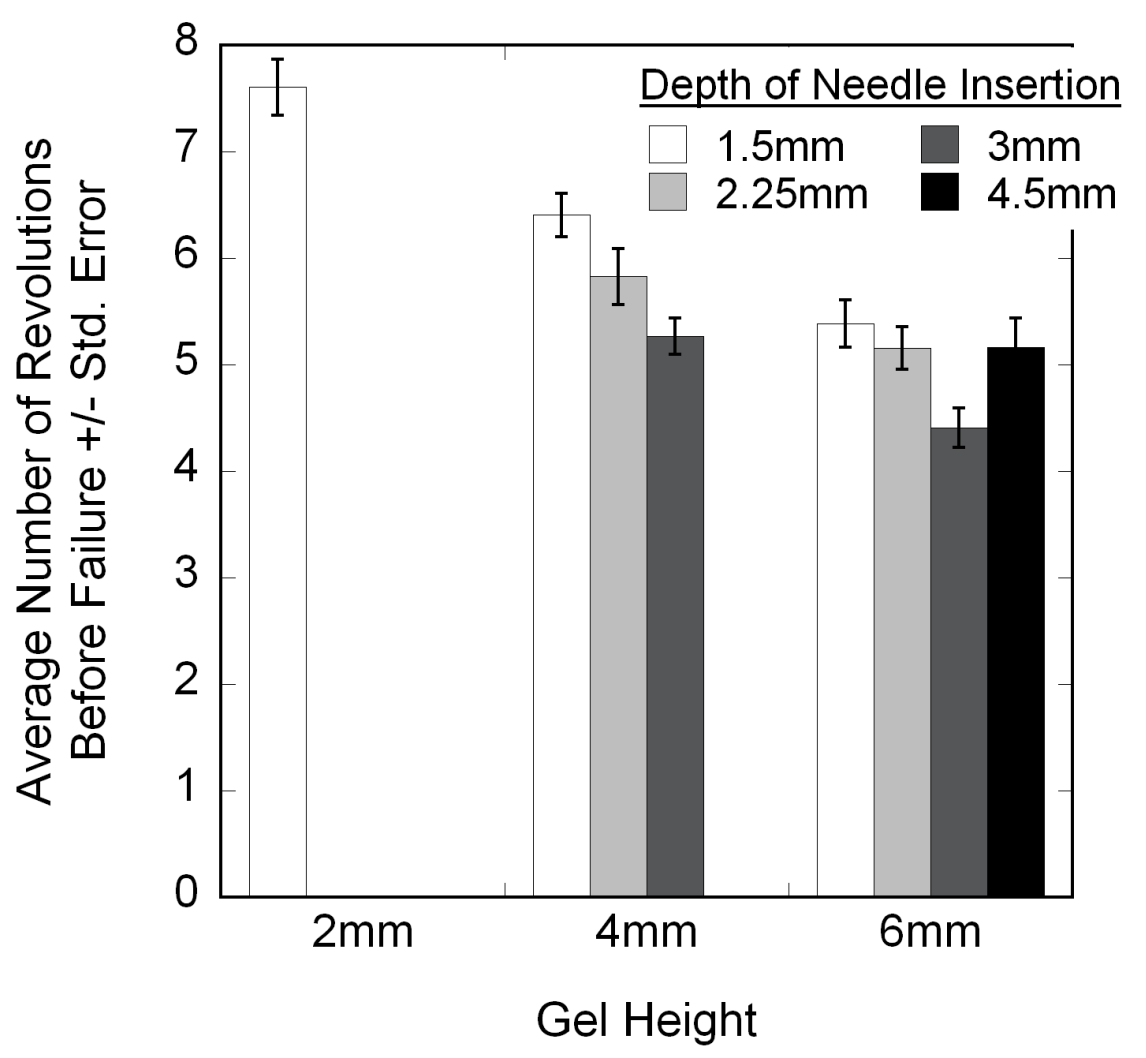
Effects of gel height and depth of needle insertion on revolutions to failure during in vitro acupuncture. Thin gels were able to withstand significantly more needling than thick ones (*P *< 0.001). For 4 mm-thick gels, revolutions to failure decreased as the depth of needle insertion increased, but this was not observed for 6 mm-thick gels.

**Figure 10:**
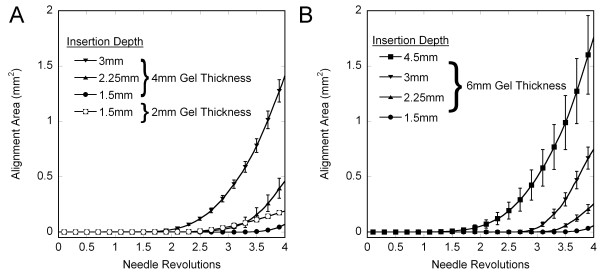
Effects of gel thickness and depth of needle insertion on the measured area of fiber alignment (average +/- std. error). (A) 2 mm-thick and 4 mm-thick gels; (B) 6 mm-thick gels. For the same depth of insertion, thin gels demonstrated more alignment than thick gels. For both 4 mm- and 6 mm-thick gels, the alignment area increased as depth of insertion increased.

The depth of insertion study was performed with a new shipment of collagen, and preliminary experiments with collagen from the crosslinking and collagen concentration studies indicated that the new batch presented significantly less alignment than the old batch, but that trends in the response were the same. As such, experiments in the depth of insertion studies were performed exclusively with the new batch of collagen and analyzed separately from the crosslinking and collagen concentration studies. Changing the depth of needle insertion significantly affected the failure of the gels (Figure 9). Thicker gels failed at fewer revolutions than thinner gels. For 4 mm-thick gels, increasing the depth/percentage of needle insertion decreased the revolutions before failure, but this was not observed consistently with the 6 mm-thick gels. The alignment of gels was also affected by insertion depth and percentage (Figure 10). In vitro acupuncture with needles inserted the same depth in gels of different thickness generated more alignment in thinner gels, indicating that the fraction of the gel that is subjected to needle rotation is an important parameter in dictating the response. Maintaining the same percentage of insertion at gels of different thickness produced greater alignment in thicker gels than thinner gels. For example, inserting a needle 3 mm into a 4 mm-thick gel and rotating the needle produced more alignment than the same procedure in a 6 mm-thick gel (inverted triangles, Figure 10A and 10B). However, inserting a needle 75% into a 6 mm-thick gel (squares, Figure 10B) produces more alignment than 75% into a 4 mm gel (inverted triangles, Figure 10A).

### Rheology measurements

**Figure 11:**
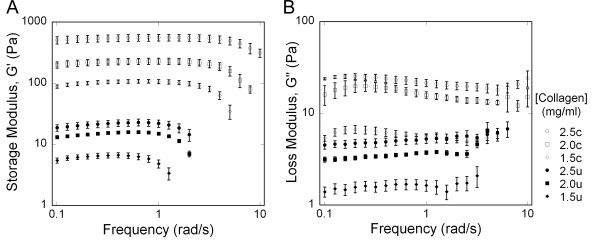
Frequency sweep of collagen gels under 1% controlled strain (average +/- standard error). (A) Storage Modulus; (B) Loss Modulus. Both the storage and loss moduli demonstrated significant increases with increasing collagen concentration and crosslinking (2-way ANOVA, *P *< 0.001). Key: c: crosslinked, u: untreated.

Storage and loss moduli were determined using parallel plate rheometry. The storage modulus showed a gradual increase with increasing frequency, before sharply dropping (Figure 11A). Inspection of gels revealed damage to the samples, which did not occur if experiments were run only at lower frequencies (data not shown), and we assumed that the damage was responsible for the apparent decrease in stiffness. In general, increased collagen concentration and crosslinking delayed this damage. The loss modulus for untreated gels showed a gradual increase at low frequencies, particularly for untreated collagen (Figure 11B). The loss modulus for crosslinked collagen decreased at moderate frequencies, and increased more sharply for all cases concurrent with the decrease in storage modulus. Two-way ANOVA revealed significant increases in the storage and loss moduli at all frequencies with increasing collagen concentration and crosslinking (all *P *< 0.001).

## Discussion

During treatment, acupuncture therapists aim to achieve "needle grasp" as a sensory marker of an appropriate degree of needle manipulation. Recent studies suggest that needle grasp occurs when collagen fibers in the subcutaneous connective tissue attach to and wind around the needle, thus imposing a local stress and strain field on the surrounding tissue [6]. In this paper, we imaged a simple, in vitro, acellular collagen gel system using polarized light microscopy during acupuncture needle rotation and measured the degree of winding in terms of fiber alignment to identify relationships between collagen concentration, crosslinking, and winding, as well as the failure of the gels.

We found that both collagen concentration and crosslinking influenced the response to controlled acupuncture needle rotation. Alignment increased with increasing collagen concentration, but decreased in gels that were crosslinked with formalin. Crosslinked gels also failed at a significantly lower number of needle rotations than untreated gels. Failure consistently occurred ~0.25 mm-1.0 mm away from the needle, and corresponded to the point where circumferential alignment of collagen fibers wound around the needle transitioned to radial alignment from fibers in the periphery of the gel being pulled into the needled area. The acute change in fiber geometry likely introduced a stress concentration that ultimately caused a tear in the collagen gel.

Altering the collagen concentration increases fiber density, which has potential indirect and direct consequences on the polarized light microscopy. First, the increase in fiber density could affect the mechanical properties simply by increasing the number of structural elements to carry load and/or by increasing fiber-fiber interactions, which would affect the tissue response to needle rotation. The increase in fiber density would also influence the degree of alignment, as measured with polarized light microscopy. An increase in fiber density implies that a greater number of fibers would be aligned for the same number of revolutions, and could therefore more efficiently rotate the polarization state of the incident light.

Crosslinking the gel decreases the flexibility of individual and aggregate fibers, which had a marked effect on the mechanical properties of the gels, increasing the storage modulus in shear by about an order of magnitude. The increased rigidity of crosslinked fibers increased the resistance to winding and deformation, and also increased the stress generated with winding, thereby leading to less alignment and earlier failure compared to untreated gels of the same concentration.

In addition to altering the mechanical properties of the gels, changing collagen concentration and crosslinking the gels could have influenced interactions and adhesion with the needle and/or the polystyrene dish. However, no gels failed at either the needle interface or the dish interface, and we believe that differences in adhesion among conditions played a minimal role in the bulk of the observed temporal response, except, perhaps, for the initial lag period in the alignment curves that represents initiation of alignment.

The differences between the response of untreated and crosslinked collagen gels to in vitro acupuncture, and particularly the earlier failure of crosslinked gels, suggests that mechanostructural differences in the soft tissues that contact an acupuncture needle during therapy may be responsible for the selective coupling and winding of collagen fibers in specific soft tissue layers during needle rotation. In vivo and explant studies have demonstrated that, although an acupuncture needle is inserted through the epidermis and dermis and into subcutaneous fat and muscle, only the subcutaneous loose connective tissue appears to specifically wind around the needle [3]. The resulting recruitment of loose connective tissue fibers towards the needle can thicken that layer and subsequently compress the overlying and underlying tissue layers, but the characteristic whorl pattern is only seen in the loose connective tissue.

The tissue properties that govern this selective adherence and winding are not yet known. However, a recent study by Iatridis et al. documented the mechanical properties in uniaxial tension of loose connective tissue from mouse explants, and noted important distinctions between loose connective tissue and other load bearing soft tissues, including skin [16]. Most soft tissues demonstrate significant non-linear stiffening above a certain strain – typically between 1% and 20%. For example, skin has a low strain modulus on the same order as loose connective tissue (2.75 kPa) up to about 10% strain [17], after which the modulus increases to ~240 kPa [17,18]. In contrast, loose connective tissue demonstrated a highly linear elastic response up to 50% strain [16]. Thus, as tissue begins to wind around the needle and deform, significantly greater stress will be generated in skin vs. loose connective tissue, and, similar to the response of our crosslinked gels, we would expect the failure stress to be reached at a lower number of revolutions. The network of collagen fibers in the dermis may be too stiff to effectively respond to acupuncture needle rotation.

In clinical acupuncture, the thickness of the connective tissue layer varies, often with the thickness of subcutaneous fat, and it is especially thick at intermuscular cleavage planes. These planes correlate anatomically to acupuncture points and meridians[14], and Langevin has shown that the resistant force to needle rotation at acupuncture points is greater than at control points, where the connective tissue layer is thinner. It was suggested that needling in locations where connective tissue is more pronounced enhances the mechanical response of fibroblasts residing within this tissue [1]. In investigating how the depth of needle insertion and gel thickness may affect the response of a homogeneous tissue, we found that both the relative depth as well as the absolute depth of insertion into the collagen gel were important factors in the failure and alignment responses. Thinner gels were able to withstand more needle rotations than thick ones. Interestingly, the failure point was reached earlier as the depth of insertion was increased in 4 mm-thick gels, but no real trend was observed in 6 mm-thick gels. For both 4 mm-thick and 6 mm-thick gels, more alignment was recorded when the depth of insertion was greater. The last observation is consistent with an increase in the number of fibers subjected to rotation via contact with the needle. We also observed that more alignment was generated for the same depth of insertion in thin gels versus thick ones. The greatest amount of alignment was observed with the greatest absolute coverage of the needle by the collagen gel – 4.5 mm insertion into a 6 mm-thick gel. We believe that the increase in thickness of collagen below the needle increases the physical resistance to drawing individual fibers up and in towards the needle, thereby creating more stress to stimulate resident cells. We also note that the tip of the acupuncture needle is tapered. The length of the tapered tip represents a greater proportion of the inserted needle at shallower insertion depths than deeper insertions. The biomechanical response of the gel, particularly the initial adhesion of the collagen fibers to the needle, may be influenced by needle diameter [14], which would be embedded in our needle depth results.

The differences with collagen concentration and crosslinking, as well as the empirical differences in alignment from separate batches of collagen (compare 2.0 mg/ml plot in Figure 6 to 3 mm insertion into 4 mm-thick gels in Figure 10A), suggest that subtle changes in tissue composition and structure may affect the biomechanical response to needle rotation in vivo, and potentially the efficacy of acupuncture therapy. It is well known that the collagen content of human skin throughout the body is non-uniform [9], and the matrix components of skin can be crosslinked, degraded, and or damaged by any number of environmental factors, including exposure to ultraviolet light, disease states, such as glycation associated with type 1 diabetes mellitus [19], and normal physiological processes, such as wound healing. There have been relatively few studies of loose connective tissue of any kind, though it is likely that the biophysical properties of this tissue also vary among individuals and with location in one individual, and may dictate, in part the efficacy of acupuncture in a particular patient or at a particular location.

It is important to keep in mind that the in vitro system developed in this work is only a first step and differs significantly from the loose connective tissue involved in acupuncture, a cellular tissue comprising primarily fibroblasts embedded in an extracellular matrix of collagen and elastin fibers and proteoglycans. We chose to begin with an acellular collagen gel, representing the most significant structural component of the extracellular matrix, to establish a baseline for further study before proceeding to the more complex cellular system, in which a number of variables can change dynamically due to fibroblast-mediated compaction, matrix synthesis, and degradation. We also chose a rotational velocity (0.3 rev/sec) significantly slower than typically applied clinically to facilitate image acquisition and reduce viscoelastic effects.

## Conclusion

The in vitro model provides a platform to study mechanotransduction during acupuncture in a highly controlled and quantitative setting. The results indicate that the mechanostructural properties of soft connective tissues may affect their response to acupuncture therapy. Based on the results of this work, the biofidelity of our in vitro system can now be systematically improved by introducing cells and additional matrix components, such as elastin and proteoglycans, to better mimic features of loose connective tissue, and by applying needle rotation protocols consistent with clinical practice. The incorporation of additional instrumentation to record the resistive torque that develops in the gel during needle manipulation and the strain within the gel would also significantly improve our ability to study the biomechanics associated with acupuncture. It is likely that the torque and strain, which may be transmitted to resident cells in vivo and in cellular assays in vitro to initiate mechanotransduction, are strongly influenced by gel or tissue composition as well as the rate and number of needle rotations. The system can then be used to aid in the determination of the quantitative biological response to biomechanical signals introduced during acupuncture needling.

## Competing interests

The authors declare that they have no competing interests.

## Authors' contributions

MJ carried out the in vitro experiments and helped draft the manuscript. LTE developed the image processing algorithms and analyzed the polarized light images. HMB and DIS conceived of the study, designed the experiments, and drafted the manuscript. All authors read and approved the manuscript.

## References

[B1] Langevin HM, Churchill DL, Fox JR, Badger GJ, Garra BS, Krag MH (2001). Biomechanical response to acupuncture needling in humans. J Appl Physiol.

[B2] Langevin HM, Churchill DL, Cipolla MJ (2001). Mechanical signaling through connective tissue: a mechanism for the therapeutic effect of acupuncture. Faseb J.

[B3] Langevin HM, Churchill DL, Wu J, Badger GJ, Yandow JA, Fox JR, Krag MH (2002). Evidence of connective tissue involvement in acupuncture. Faseb J.

[B4] Langevin HM, Konofagou EE, Badger GJ, Churchill DL, Fox JR, Ophir J, Garra BS (2004). Tissue displacements during acupuncture using ultrasound elastography techniques. Ultrasound Med Biol.

[B5] Langevin HM, Bouffard NA, Badger GJ, Churchill DL, Howe AK (2006). Subcutaneous tissue fibroblast cytoskeletal remodeling induced by acupuncture: evidence for a mechanotransduction-based mechanism. J Cell Physiol.

[B6] Langevin HM, Bouffard NA, Churchill DL, Badger GJ (2007). Connective tissue fibroblast response to acupuncture: dose-dependent effect of bidirectional needle rotation. J Altern Complement Med.

[B7] Da Silva DFT, Vidal BC, Zezell DM, Zorn TMT, Nunez SC, Ribeiro MS (2006). Collagen birefringence in skin repair in response to red polarized-laser therapy. Journal of Biomedical Optics.

[B8] Pierce MC, Strasswimmer J, Park BH, Cense B, de Boer JF (2004). Birefringence measurements in human skin using polarization-sensitive optical coherence tomography. Journal of Biomedical Optics.

[B9] Vitellaro-Zuccarello L, Cappelletti S, Dal Pozzo Rossi V, Sari-Gorla M (1994). Stereological analysis of collagen and elastic fibers in the normal human dermis: variability with age, sex, and body region.. The Anatomical Record.

[B10] Debessa CRG, Maifrino LBM, de Souza RR (2001). Age related changes of the collagen network of the human heart. Mechanisms of Ageing and Development.

[B11] Mays PK, McAnulty RJ, Campa JS, Laurent GJ (1995). Age-related Alterations in Collagen and Total Protein Metabolism Determined in Cultured Rat Dermal Fibroblasts: Age-related Trends Parallel those Observed in Rat Skin In Vivo. Int J Biochem Cell Biol.

[B12] Vogel HG (1980). Influence of maturation and aging on mechanical and biochemical properties of connective tissue in rats. Mech Ageing Dev.

[B13] Takahashi M, Hoshino H, Kushida K, Inoue T (1995). Direct Measurement of Crosslinks, Pyridinoline, Deoxypyridinoline, and Pentosidine, in the Hydrolysate of Tissues Using Hign-Performance Liquid Chromatrography. Analytical Biochemistry.

[B14] Langevin HM, Yandow JA (2002). Relationship of acupuncture points and meridians to connective tissue planes. Anat Rec.

[B15] Shreiber DI, Enever PAJ, Tranquillo RT (2001). Effects of PDGF-BB on rat dermal fibroblast behavior in mechanically stressed and unstressed collagen and fibrin gels. Experimental Cell Research.

[B16] Iatridis JC, Wu J, Yandow JA, Langevin HM (2003). Subcutaneous tissue mechanical behavior is linear and viscoelastic under uniaxial tension. Connect Tissue Res.

[B17] Eshel H, Lanir Y (2001). Effects of strain level and proteoglycan depletion on preconditioning and viscoelastic responses of rat dorsal skin. Ann Biomed Eng.

[B18] Oxlund H, Manschot J, Viidik A (1988). The role of elastin in the mechanical properties of skin. J Biomech.

[B19] Vishwanath V, Frank KE, Elmets CA, Dauchot PJ, Monnier VM (1986). Glycation of skin collagen in type I diabetes mellitus. Correlation with long-term complications. Diabetes.

